# Assessment of glyphosate and its metabolites’ residue concentrations in cultured African Catfish offered for sale in selected markets in Ibadan, Oyo State, Nigeria

**DOI:** 10.3389/ftox.2023.1250137

**Published:** 2023-11-02

**Authors:** Selim Adewale Alarape, Adekemi Florence Fagbohun, Oladeni Adegoke Ipadeola, Anthony Ayodeji Adeigbo, Ridwan Olamilekan Adesola, Olanike Kudirat Adeyemo

**Affiliations:** ^1^ Department of Veterinary Public Health and Preventive Medicine, Faculty of Veterinary Medicine, University of Ibadan, Ibadan, Nigeria; ^2^ Federal College of Animal Health and Production Technology, Ibadan, Nigeria; ^3^ Department of Veterinary Medicine, Faculty of Veterinary Medicine, University of Ibadan, Ibadan, Nigeria

**Keywords:** glyphosate, *Clarias gariepinus*, liquid chromatography, residue, metabolites, herbicides

## Abstract

**Introduction:** Glyphosate is a non-targeted organophosphate insecticide whose solubility and mobility in hydrophilic solvents enable its rapid leaching into the soil and subsequent contamination of ground and surface water and possible build-up in the aquatic food chain. Based on the public health importance of glyphosate in fish through consumption, it is crucial to determine the current residue concentration in culture *Clarias gariepinus* species. The aim of the present study is to evaluate glyphosate’s residue concentrations and its metabolites in cultured African Catfish offered for sale in selected markets in Ibadan.

**Methods:** A total of twenty-five (25) adult *Clarias gariepinus* (300 ± 50 g) were sourced from five (5) selected active fish markets (Ojoo, Iwo road, Eleyele, Challenge, and Apata) within the Ibadan metropolis. The collected fish tissue samples (liver, kidney, and spleen) were prepared for glyphosate residue concentration analysis using Liquid Chromatography (LC).

**Results:** The results showed that glyphosate residues were recorded in all the seventy-five (75) fish tissue samples obtained from the selected fish markets in the Ibadan metropolis and all residue concentrations were above both the recommended Acceptable Daily Intake (ADI) of 1.0 mg/kg (1 × 10^−3^ mg/L) and Maximum Residue Limits (MRL) of 0.01 mg/kg (1 × 10^−5^ mg/L). Isopropylamine has the highest residue concentration followed by N-Phosphonomethyl and Aminomethylphosphonic Acid (AMPA), while N-Acetyl Glyphosate has the least residue concentration across the sampled markets.

**Discussion:** The presence of residues of glyphosate and its metabolites in ready-to-eat fish calls for holistic, systematic, and effective risk management strategies towards monitoring pesticide/herbicide usage in aquaculture production and ensuring the provision of wholesome fish and fish products for the consumers.

## Introduction

Fish farming is a popular industry in Nigeria with increased demand for nutritious animal protein. Catfish farming is practiced by around 80% of fish farmers in Nigeria, and it is the most popular variety of fish employed in aquaculture. As a result, the usage of agrochemicals for fish growth and sustainability has increased. On farms, various agrochemicals such as Formalin, Copper Sulphate, Malachite green, and Potassium Permanganate among others have been abused and/or misused (Adeyemo et al., 2011; Alarape et al., 2013). The adverse side effects of glyphosate and aminomethyl phosphonic acid, or metabolites, on soil, water quality, plant, animal, and human health have been studied extensively in recent years due to glyphosate’s extensive use and accumulation in the environment and food products (Battaglin et al., 2014; Séralini et al., 2014). In 2015, the World Health Organization (WHO) categorized the glyphosate herbicide as possibly human carcinogenic based on their most recent findings on its potential chronic negative effects (EFSA, 2015; Guyton et al., 2015; IARC, 2015).

According to the Rodriguez-Gil et al. (2017) report, a typical and maximum application of glyphosate and runoff rates from the soil resulted in estimated concentrations of 0.21–0.99 mg POEA L−1 surface water, which were responsible for the estimated impairment of 21%–43% of a wide range of aquatic species. Sandrini et al. (2013) found that pure glyphosate suppressed acetylcholinesterase activity in brown mussels (*Perna perna*) and several fish species at low concentrations (1–676 mg L−1) (Menéndez-Helman et al., 2012; Sandrini et al., 2013). These findings are consistent with the effects of glyphosate on terrestrial animals. In zebrafish embryos exposed to Roundup^®^ at higher concentrations (50 mg L−1), developmental (forebrain, midbrain, and eye) damage was seen. Glyphosate was reported to have damaged the zebrafish primary motoneurons at a low concentration of 0.01 mg L−1 resulting in erratic movements at a young age (Roy et al., 2016; Zhang et al., 2017a). The metabolism in many tissues was impacted by the prolonged exposure of goldfish (*Carassius auratus*) to glyphosate (34 mg L−1), which resulted in an excess of oxidative stress and severe kidney damage (Li et al., 2017). The interactions between fish and their diseases are also impacted by glyphosate, in addition to the direct effects on aquatic creatures mentioned above.

Herbicides made of glyphosate may pollute the soil in and surrounding treated regions. Because of its delayed breakdown by soil microbes due to its adsorption to clay and organic matter, which causes its accumulation in soils over time, glyphosate was initially not thought to be an issue for both ground and surface water (Sihtmäe et al., 2013; Banks et al., 2014; Sviridov et al., 2015; Travaglia et al., 2015; Cassigneul et al., 2016; Okada et al., 2016; Sidoli et al., 2016). By multiple earlier observations, glyphosate and metabolites, its breakdown product, may linger for more than a year in soils with a high clay concentration, but swiftly wash out of sandy soils (Bergström et al., 2011; Okada et al., 2016; Sidoli et al., 2016). Despite its attachment to clay and organic matter, glyphosate and metabolites are degraded in part by soil pH (Zhang et al., 2015b), and after a significant amount of rain, some of the chemicals dissolve in groundwater (Maqueda et al., 2017). The soil particles carrying glyphosate and metabolites are also transported into surface water by rain and erosive processes, where they can either persist in the particulate phase or dissolve (Maqueda et al., 2017).

While contaminated particles might settle and mix with the bottom sediment, dissolved glyphosate and metabolites in surface water can sorb down to the bottom (Maqueda et al., 2017). Due to its widespread acceptance, glyphosate and its metabolites are currently present in large quantities in a range of natural waterways and sediments. Glyphosate biodegrades much more slowly in sediment than in water (Grandcoin et al., 2017; Maqueda et al., 2017; Poiger et al., 2017). In the United States, where glyphosate-resistant crops are produced on genetically engineered plants, glyphosate and metabolites are commonly found in soil, surface water, and groundwater (Battaglin et al., 2014). Glyphosate levels in the river and stream water have been found to range from 2 to 430 g L−1 (Coupe et al., 2011; Mahler et al., 2017). Additionally, it has been found in the spring snowmelt, air, and rain during the planting and growing season (Chang et al., 2011). When glyphosate is used, it eventually winds up in seawater, where it is extremely persistent (Mercurio et al., 2014).

While growing genetically modified crops is prohibited in Europe, unlike the United States, glyphosate has been found in several water sources (although at lower concentrations than in the United States) with very low concentrations (0.1–2.5 g L−1) surface water samples in Germany (Skark et al., 1998), northeastern Spain (Sanchis et al., 2012), Hungary (Mörtl et al., 2013), France (Villeneuve et al., 2011), and Switzerland (Poiger et al., 2017). In addition to runoff from agricultural land, Grandcoin et al. (2017) and Hanke et al. (2010) suggest that urban runoff is another source of glyphosate for streams and rivers. According to Rosenbom et al. (2010), numerous Northern European nations have outlawed the use of glyphosate on paved surfaces because it increases runoff from impermeable and linked paved surfaces. Yet glyphosate and metabolites were discovered in samples of sewage and stormwater runoffs, outputs from wastewater treatment plants, and even packaged bottled water (Birch et al., 2011; Grandcoin et al., 2017). According to reports, glyphosate and metabolites are typically found in drinking water, however at very low amounts that are below the recommended daily intake (ADI) that was established in 1997 (WHO, 2005).

Although, Bastos et al. (2020) reprted that there were no published works that have evaluated the toxicological effects of glyphosate in aquatic mammals and birds which demonstrated lack of knowledge on the risk of exposure of aquatic animal in the environments contaminated by glyphosate. But in contrary, Shehata et al. (2014), treported that glyphosate residues could be found in the liver, spleen, lung, intestine, heart, muscles, kidney, and animal feed. This is because glyphosate is applied at higher rates and more frequently than before. Eighty-nine percent (89%) of the corn crop and 94% of the soybean crop in the United States were herbicide-tolerant varieties, and glyphosate was likely sprayed on most of them. Additionally, a considerable portion of the feed used for animals comes from genetically modified crops. According to research, 57% of maize and 85% of soybeans are used annually in livestock diets worldwide (McNaughton et al., 2011). In none of the aforementioned research was it discovered that feeding animals glyphosate-sprayed crops during cultivation decreased their output. In the findings of Muhammad et al. (2021), they concluded that a relatively high concentration of technical grade glyphosate is needed to induce significant changes in fish, however, it was stated that the bodyweight index is the most sensitive toxicity parameter in that at 25 mg/L of glyphosate, there was reduction in the fish body weight. Negative correlations between the glyphosate concentration and toxicity parameters such as specific growth rate (SGR), hepato-somatic index (HIS), and gonado-somatic index (GSI) were observed (Muhammad et al., 2021). Ruminants are major consumers of genetically modified (GE) crops, and as bacterial protein and end products of microbial fermentation make up a sizable amount of their metabolizable nutrients, they serve as models for research on the impact of pesticide residues on gut microbes. Similar to this, although microbial proteins are not consumed, bacterial fermentation activity in the hindguts of non-ruminant animals is significant for some nutrients. Recently, glyphosate exposure toxicity has been reported to cause several changes such as haematologic and biochemical processes in tissues (Modesto and Martinez, 2010), genotoxicity (Guilherme et al., 2012; de Brito Rodrigues et al., 2019), histopathological damage and immunotoxicity (Antunes et al., 2017; Ma et al., 2019), or cardiotoxicity (Gaur and Bhargava, 2019) in fish.

This present study was designed to evaluate the residue concentrations of glyphosate and its metabolites in cultured African Catfish offered for sale in selected markets in Ibadan.

## Materials and methods

### Sample collection

Five adults *Clarias gariepinus* samples (300 ± 50 g) each were sourced from five selected active cultured fish markets (Ojoo, Iwo road, Eleyele, Challenge, and Apata) within the Ibadan metropolis. A total number of twenty-five adult *Clarias gariepinus* were transported to the Fish and Wildlife Unit Laboratory, Department of Veterinary Public Health and Preventive Medicine, Faculty of Veterinary Medicine, University of Ibadan.

Each fish was pithed before sacrifice. Organs (muscle, liver, and kidney) were then harvested through dissection of the fish and separately put inside the sterile sample nylons, sealed, and kept under the ice. The collected samples were then taken to the International Institute of Tropical Agriculture (I.I.T.A) for glyphosate residue concentration analysis.

### Solid-phase extraction (SPE) and quantification

The SPE was carried out based on the procedures described by Delmonico et al. (2014). The digestion tubes were washed with water, later rinsed with distilled water, and finally rinsed using 0.5% HCl in ultra-pure water (purest scientific water) and allowed to dry. The fish tissue samples were homogenized and stored in Pyrex tubes in a viscous form. 0.5 mL of homogenized samples were poured into each of the digestion tubes using a precision pipette. About 20 mL of the ammomethyl-phosphonic-nitric acids mixture in the ratio 5:2:5 was then dispensed into each of the digestion tubes containing the samples. The mix is then digested for two and half hours on a digestion block at 25°C and covered with a condenser.

At the end of the digestion process, the digests were brought out and allowed to cool to room temperature (25°C). Each cooled digest was made up to 25 mL volumes with ultra-pure water, covered with paraffin paper, swirled to mix properly, tightly covered, and shaken on a mechanical shaker for 10 min. They were then centrifuged for 5 min at 5,000 revolutions per minute (rpm). The resultant supernatants were analyzed using Reverse Phase LC (Agilent Technologies, Santa Clara, CA, United States) with C18, 5 µm 120 Â, 4.6 × 250 mm column as stationary phase and methanol/H_2_O (90:10) as mobile phase at a flow rate of 1 mL/min for 30 min.

## Method validation

The SPE-LC method’s specificity, linearity, sensitivity, accuracy, precision, and resistance to matrix effects were all validated.

### Data analysis

Data obtained were calculated by the interphase software with LC. The results were expressed in frequency, percentages, and variance (descriptive statistics) on SPSS Statistics Version 26.

### Ethical consideration

The University of Ibadan Animal Care and Use in Research Committee accepted the research protocol, which was given the number UI-ACUREC/019-0220/6.

## Results

The residue concentrations of glyphosate its metabolites as detected in fish organs and muscles from different fish markets are shown in Supplementary Figures S1–S15. Isopropylamine has the highest residue concentration followed by N-Phosphonomethyl and Aminomethylphosphonic Acid (AMPA), while N-Acetyl Glyphosate has the least residue concentration in fish organs and muscles obtained from Ojoo fish market (Supplementary Figures S1–S3).

From the ANOVA result obtained in Table 1, there is significance difference (*p*-value less than 0.05) among the fish organs and muscles across the glyphosate and its metabolites.

**TABLE 1:** Comparison of glyphosate and its metabolites’ residue concentrations in organs and muscles of cultured African Catfish offered for sales at different fish markets in Ibadan.

Markets	Organs	*p*-value ( ≤0.05 )
Ojoo	Liver	0.0006
	Kidney	
	Muscle	
Eleyele	Liver	0.01
	Kidney	
	Muscle	
Apata	Liver	0.01
	Kidney	
	Muscle	
Challenge	Liver	0.01
	Kidney	
	Muscle	
Iwo Road	Liver	0.007
	Kidney	
	Muscle	

**FIGURE 1:**
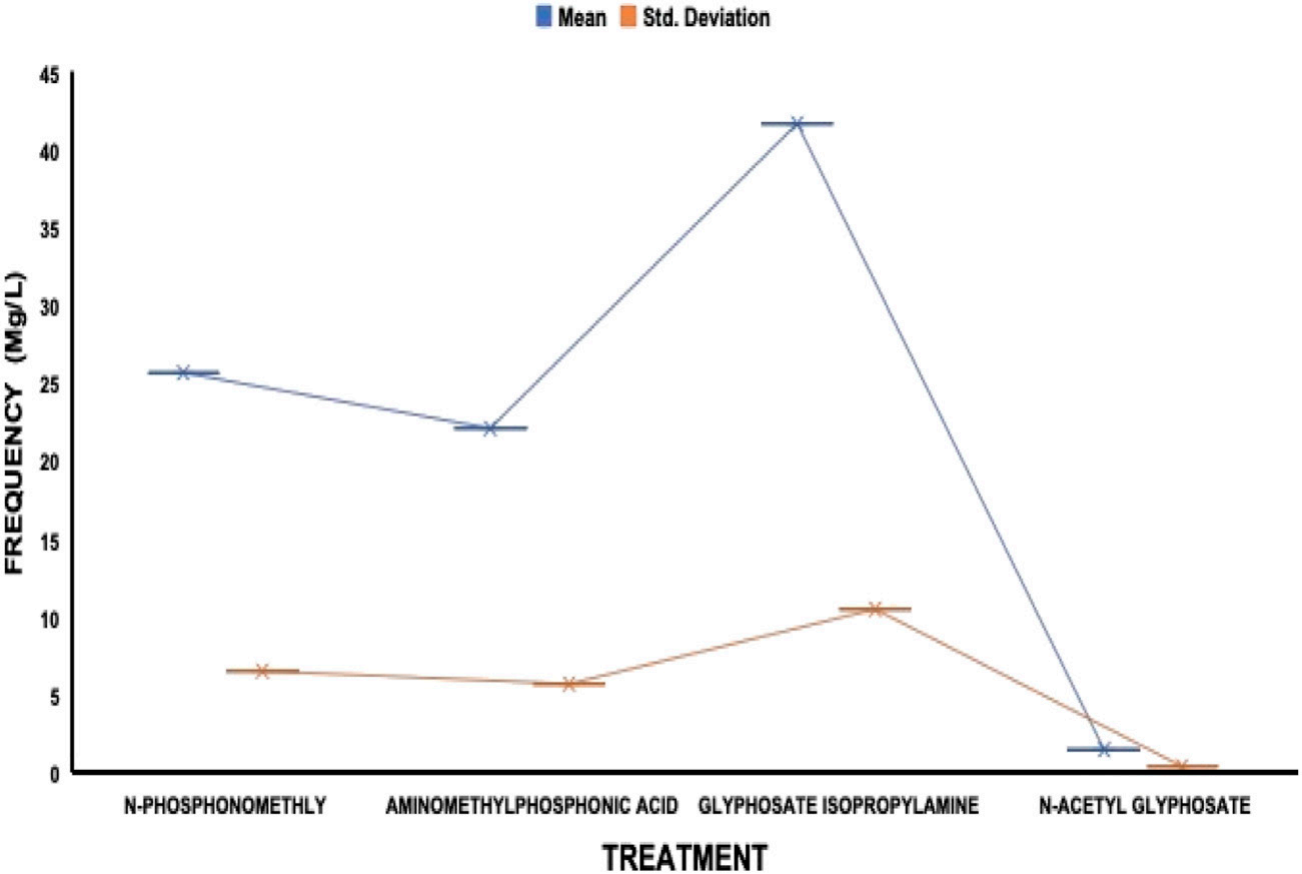
Frequency of glyphosate and its metabolites’ residue concentrations present in the Catfish organs and muscles offered for sale at Ojoo fish market. The error bars of box plots represent mean and standard deviation.

**FIGURE 2:**
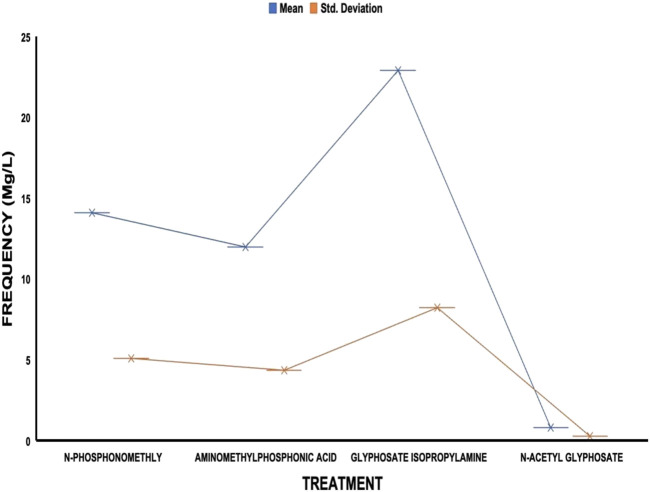
Frequency of glyphosate and its metabolites’ residue concentrations present in the Catfish organs and muscles offered for sale at Iwo road fish market. The error bars of box plots represent mean and standard deviation.

**FIGURE 3:**
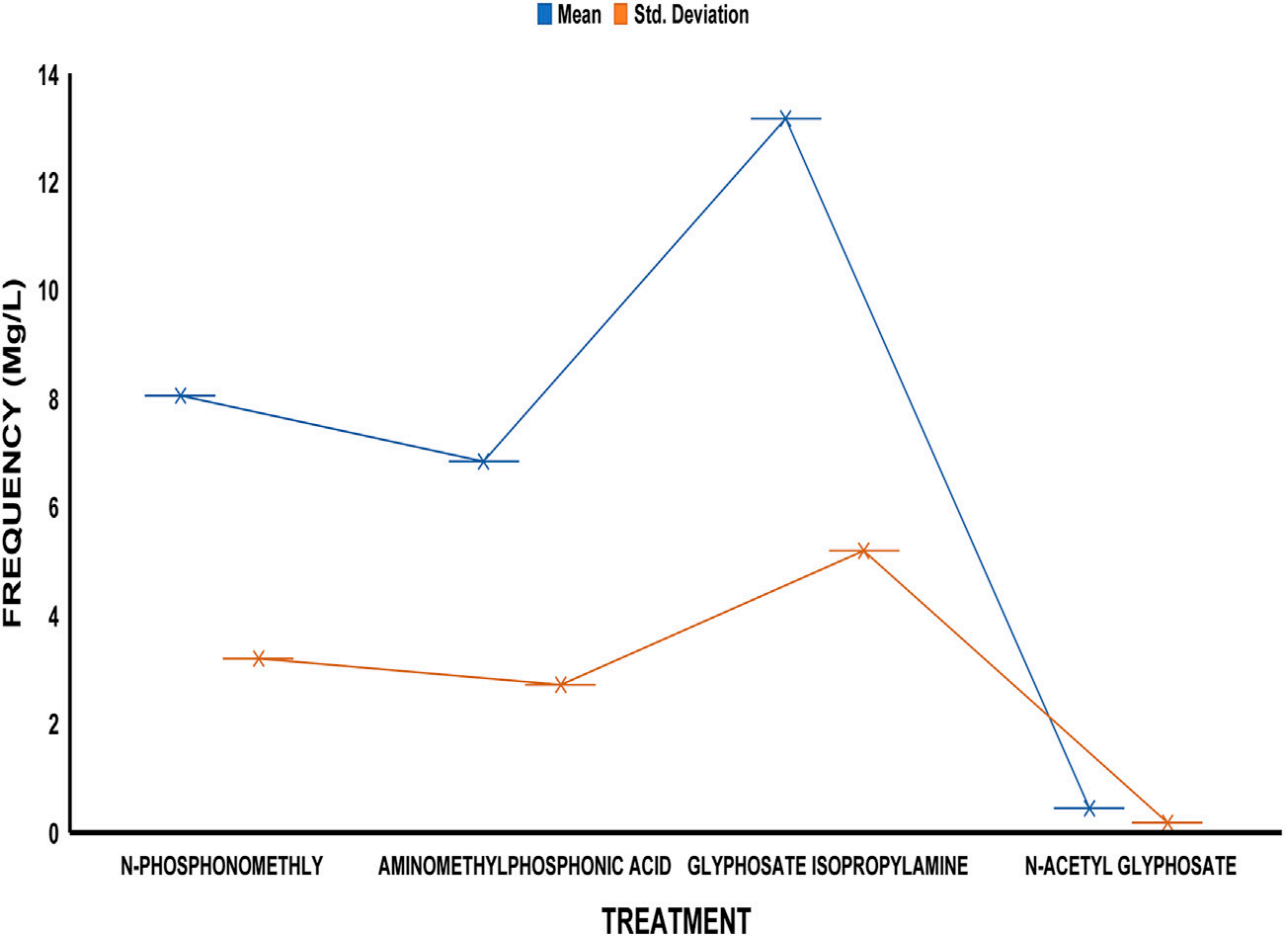
Frequency of glyphosate and its metabolites’ residue concentrations present in the Catfish organs and muscles offered for sale at Eleyele fish market. The error bars of box plots represent mean and standard deviation.

**FIGURE 4:**
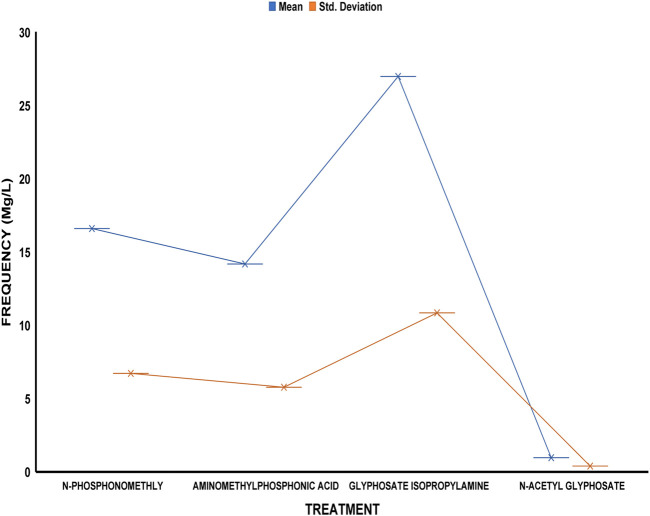
Frequency of glyphosate and its metabolites’ residue concentrations present in the Catfish organs and muscles offered for sale at Apata fish market. The error bars of box plots represent mean and standard deviation.

**FIGURE 5:**
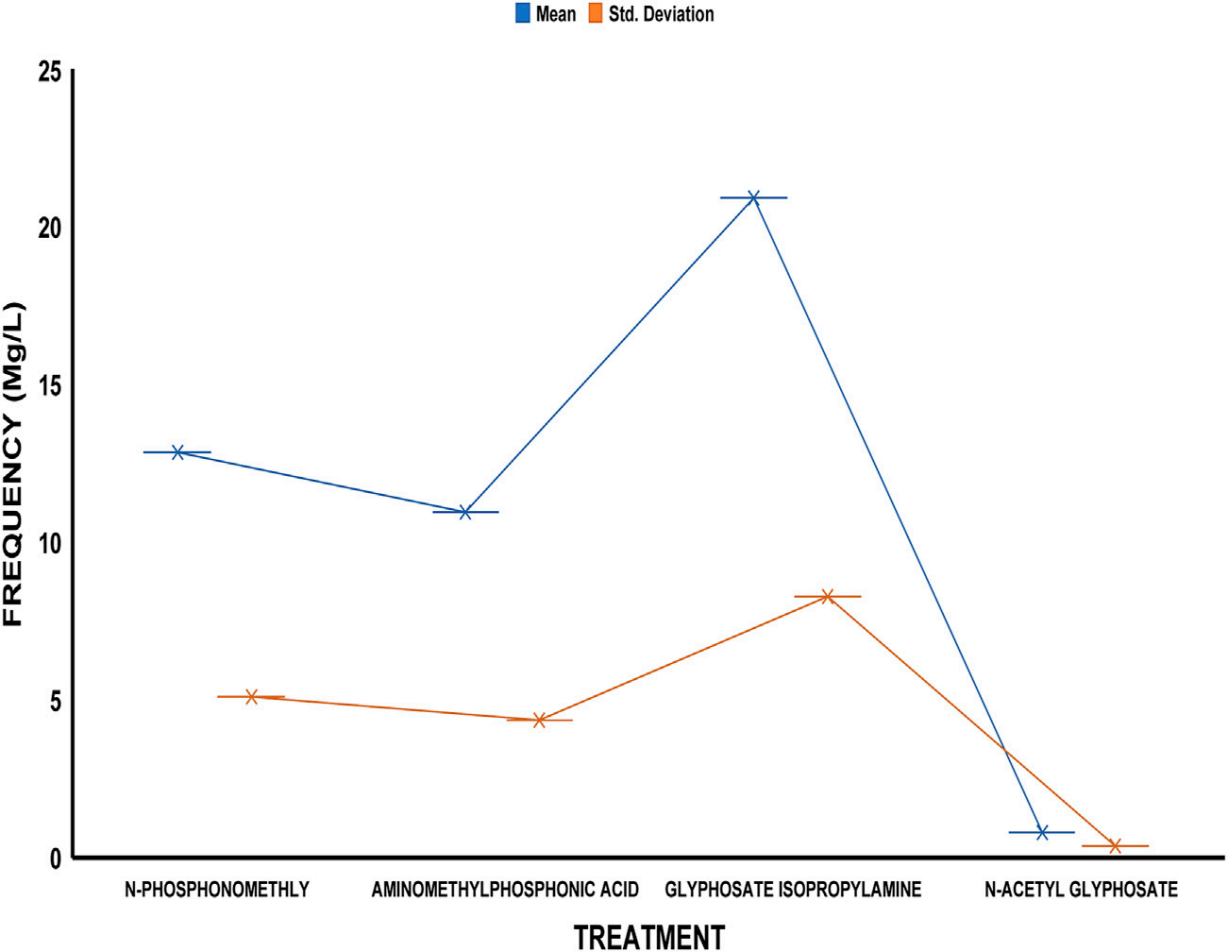
Frequency of glyphosate and its metabolites’ residue concentrations present in the Catfish organs and muscles offered for sale at Challenge fish market. The error bars of box plots represent mean and standard deviation.

The average residue concentrations of glyphosate and its intermediates as detected in the fish organs and muscles samples (liver, kidney, and muscle) were shown in Figures 1–5. Isopropylamine has the highest statistically significant residue concentration (*p*-value ≤0.05) followed by N-Phosphonomethyl and Aminomethylphosphonic Acid (AMPA), while N-Acetyl Glyphosate has the least residue concentration in fish organs and muscles obtained across all the fish market (Figures 1–5). Muscle had the highest residue concentration of both glyphosate and its metabolites across all the fish markets. Glyphosate and its metabolites residue concentration detected in Kidney were lower than those detected in muscle and higher than the detected concentrations in the liver samples across all the fish markets (Figures 1–5).

Isopropylamine has the highest residue concentration followed by N-Phosphonomethyl and Aminomethylphosphonic Acid (AMPA), while N-Acetyl Glyphosate has the least residue concentration in fish tissues obtained across all the fish market (Figures 1–5).

## Discussion

Large quantities of glyphosate-based herbicides are applied to crops two to three times per season to get rid of weeds that grow back after herbicides application. Glyphosate residues can remain stable in foods for years, irrespective of the method of food preservation. The health of both humans and animals may be impacted by glyphosate, according to several earlier research. In this study, glyphosate residues were recorded in all the fish samples obtained from the selected fish markets in the Ibadan metropolis which is in agreement with the reports of Kruger et al. (2013). The detection of glyphosate residues in the fish organs and muscles in this study confirmed the use of several glyphosate-based herbicides (Force-Up, Vinash, Para force, and Round-Up) on the fish farms (fish as a non-target organism) in Nigeria (Alarape et al., 2021), use of banned pesticides like pyrethroids, organophosphates, carbamates, and organochlorine in agricultural products in Bangladesh (Jallow et al., 2017) and Ghana (Essumang et al., 2009). Although glyphosate-based herbicides were not used directly on fish, environmental contamination may be a possible source of the herbicide residues found in fish in the research location where such herbicides are used on farms close to fish farms. In addition to demonstrating that glyphosate and its metabolites were present in the fish sold for human consumption, it also demonstrated a definite pattern regarding the degree of chemical contamination in the fish farms’ water supplies. This is supported by a 2009 investigation by Essumang et al. (2009) that found variable concentrations of organochlorine (OC) and organophosphorus (OP) pesticides in the lagoons of Etsii, Fosu, Korle, and Chemu in Ghana.

Glyphosate and several of its metabolites (AMPA, N-acetyl glyphosate, and N-acetyl AMPA) have an acceptable daily intake (ADI) for people of 1.0 mg/kg (1 × 10^−3^ mg/L) (FAO, 2004; FAO, 2011). The findings of this investigation showed that the residue of glyphosate and its metabolites in the fish samples exceeded recognized safety thresholds. The environmentally relevant concentration of glyphosate is about 0.4 mg/L (Glusczak et al., 2007). Despite this, studies based on actual water runoffs from fields freshly applied with glyphosate formulation have demonstrated glyphosate concentrations as high as 5.2 mg/L (Edwards et al., 1980), whilst simulated studies using sand as a matrix showed water runoffs containing as high as 17 mg/L glyphosate (Fu, 2020). According to Vicini et al. (2021), the permitted concentration in drinking water in Europe is less than 0.1 mg/L, but the maximum residue limit (MRL) as suggested by WHO and FAO [Codex, Alimentarius Commission (2009)] was 0.01 mg/kg (1 × 10^−5^ mg/L) (EPA, 2015). In this study, all fish samples obtained from the fish markets in Ibadan had glyphosate and its metabolites residue concentration above the recommended MRL. Glyphosate Isopropylamine had the highest residue concentration in all the fish samples analyzed while N-Acetyl Glyphosate had the least residue concentration (Figures 1–5). The highest glyphosate and its metabolites’ residue concentration was recorded throughout the fish muscle samples, followed by the fish kidney while the liver had the least residue concentrations when compared with both muscle and kidney (Supplementary Figures S1–S15).

The findings of Akan et al. (2019) are in contrast to the high glyphosate residue concentrations reported in this study, where the concentrations of herbicide residues recorded were below both the WHO and FAO maximum residue limit (MRL) of 0.01 mg/kg (1 × 10^−5^ mg/L) and the acceptable daily intake value (ADI) of 0.006 mg/kg (6 × 10^−6^ mg/L), which is considered safe for consumption as of the time of their present research work. The difference in the findings may be due to differences in vegetation, weather, and rates of usage of herbicides in the Northern part of Nigeria. When animals are fed crops cultivated with glyphosate or indirectly exposed to glyphosate, it is not anticipated that animal products, except kidney and liver due to their physiological functions, will contain appreciable residues of glyphosate. This is because glyphosate has a high water solubility (10.5 g/L), a low octanol-water partition constant (log POW = 3.2), and is quickly excreted through the kidney (Bus, 2015). This assertion is in contrast to the findings in this study because glyphosate residues were detected in the muscles of the fish sample with the highest concentrations.

## Conclusion

The increased use of herbicides in agricultural sectors is a result of the world’s rising food demand, but their residues in agricultural products, particularly fish, are raising serious health issues for consumers and public health officials because they are linked to food safety. The presence of a high residue concentration of glyphosate and its metabolites in the muscles of collected fish samples calls for direct and targeted regulations on its use in food animal-based farms.

Despite the advantages of using glyphosate-based herbicides, the results of this study and many others show that these chemicals can have negative effects on the aquatic environment, human health, and even the global food-chain cycle (Abdulkareem et al., 2014). Through the ingestion of feed and contact with the usage of polluted water, glyphosate residue may come into contact with humans and animals. Singh et al. (2020) claim that the extensive use of glyphosate has caused ecosystems to become contaminated, which is negatively affecting microorganisms, plants, animals, and humans. Therefore, this study has established the presence of glyphosate-based herbicide and its metabolites in cultured *Clarias gariepinus* (African Catfish) offered for sale in fish markets within the Ibadan metropolis.

## Data Availability

The original contributions presented in the study are included in the article/Supplementary Material, further inquiries can be directed to the corresponding author.
