# Supplementary material for: Assessment of glyphosate and its metabolites’ residue concentrations in cultured African Catfish offered for sale in selected markets in Ibadan, Oyo State, Nigeria
Source: Front Toxicol. 2023 Nov 2;5:1250137. doi: 10.3389/ftox.2023.1250137 (PMC10653321; doi:10.3389/ftox.2023.1250137)
Supplement: Supplementary file 6 [file Image13.pdf]

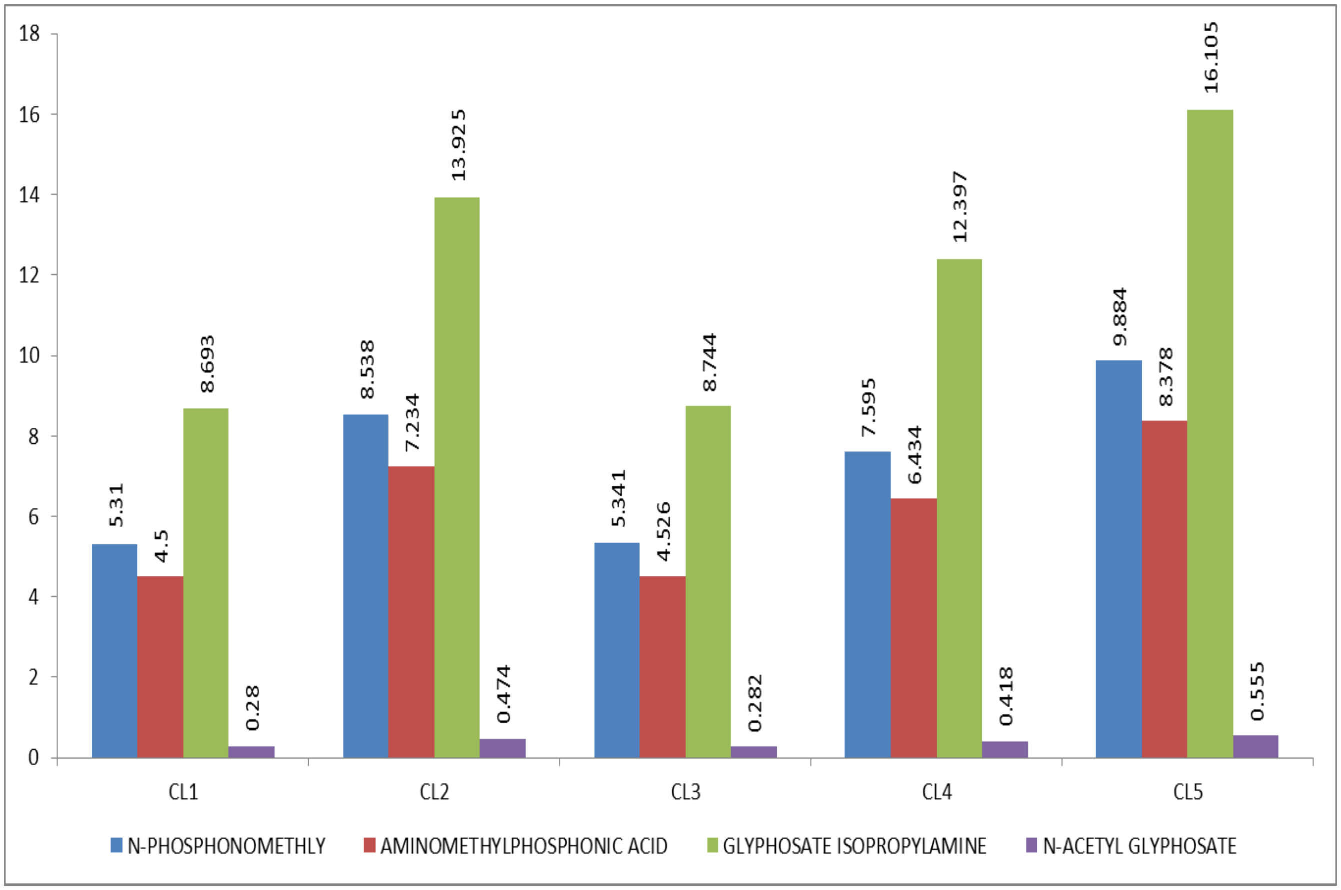

Figure 13. Residues concentrations (mg/L) of Glyphosate and its metabolites present in the Livers of Catfish offered for sale in Challenge Fish Market
